# Scale-aware dense residual retinal vessel segmentation network with multi-output weighted loss

**DOI:** 10.1186/s12880-023-01061-y

**Published:** 2023-07-29

**Authors:** Jiwei Wu, Shibin Xuan

**Affiliations:** 1 School of Artificial Intelligence, Guangxi Minzu University, Daxue East Road 188, Nanning, China; 2 Guangxi Key Laboratory of Hybrid Computation and IC Design Analysis, Nanning, China

**Keywords:** Retinal vessel segmentation, U-shape network, Deep learning

## Abstract

**Background:**

Retinal vessel segmentation provides an important basis for determining the geometric characteristics of retinal vessels and the diagnosis of related diseases. The retinal vessels are mainly composed of coarse vessels and fine vessels, and the vessels have the problem of uneven distribution of coarse and fine vessels. At present, the common retinal blood vessel segmentation network based on deep learning can easily extract coarse vessels, but it ignores the more difficult to extract fine vessels.

**Methods:**

Scale-aware dense residual model, multi-output weighted loss and attention mechanism are proposed and incorporated into the U-shape network. The model is proposed to extract image features through residual module, and using a multi-scale feature aggregation method to extract the deep information of the network after the last encoder layer, and upsampling output at each decoder layer, compare the output results of each decoder layer with the ground truth separately to obtain multiple output losses, and the last layer of the decoder layers is used as the final prediction output.

**Result:**

The proposed network is tested on DRIVE and STARE. The evaluation indicators used in this paper are dice, accuracy, mIoU and recall rate. On the DRIVE dataset, the four indicators are respectively 80.40%, 96.67%, 82.14% and 88.10%; on the STARE dataset, the four indicators are respectively 83.41%, 97.39%, 84.38% and 88.84%.

**Conclusion:**

The experiment result proves that the network in this paper has better performance, can extract more continuous fine vessels, and reduces the problem of missing segmentation and false segmentation to a certain extent.

## Background

The changes in the geometric characteristics of retinal vessels are closely related to the health status of patients, which can provide a good reference for the diagnosis of diabetes. Retinal vessels segmentation is one of the common tasks in vessels segmentation, retinal vessels contain rich geometric features, such as the length and angle of the branch. These geometric features reflect the patient’s own health status and clinical manifestations, and can diagnose many diseases. Through correct identification and diagnosis, it can provide timely reference for the treatment of eye diseases [1, 2].

The blood segmentation is divided into manual segmentation and automatic segmentation. Manual segmentation of vessels requires high professional level of operators, only be applied in some specific fields. The early methods are mainly based on traditional image processing and unsupervised learning methods, such as morphology, wavelet, clustering, etc, which has certain effect in segmentation task, but it is greatly affected by noise, so that it is difficult to meet the needs of development.

At present, most image segmentation methods are based on deep learning, because they have stronger feature extraction ability and better performance. The full convolutional neural network (FCN) [3] is a early and widely used semantic segmentation network. This model replaces all the full connection layers with convolutional layers, which can adapt to any size of input, and combines structures of different layers. UNet [4] is a variant of FCN network, belongs to encoder-decoder structure, which is commonly used for medical image segmentation. By using the skip connetction, the spatial information of the encoder can be transmitted to the decoder, and more dimensional and location information can be retained. Now UNet has become the basic network for most medical image segmentation. SA-UNet [5] optimized based on the UNet structure improved the performance of retinal vessel segmentation by adding spatial attention modules.

FCN and UNet have become very commonly used in image segmentation. However, as the number of layers increases, optimization problems such as gradient explosion and vanishing will arise, making network training difficult. With the proposal of deep residual neural network [6], the above problems have been well solved. Weighted Res-UNet [7] improves the accuracy of the network by combining the original UNet with the residual structure. ResUNet++ [8] is optimized on the basis of the ResNet and U-shape Net, and performs well in polyp segmentation, by adding squeeze and excitation block, adaptive spatial pyramid pooling(ASPP) and other methods. DR-Vnet [9] optimized on the basis of UNet, modified the residual convolution module into a residual dense-net block, and combined with the residual squeeze and excitation block, greatly improves the segmentation accuracy.

However, the skip connection will transfer all information from the encoder layers to the decoder layers, meanwhile include irrelevant background information, which will affect the segmentation performance. Therefore, attention mechanism is introduced to inhibit irrelevant features in training. The essence of attention mechanism is weighting, which highlights the features of certain regions. Attention U-Net [10, 11] combining UNet and attention gates improved the medical image segmentation accuracy without introducing additional positioning modules. Hard Attention Net [12] uses different attention mechanisms to divide input images into different regions, and then combines the features from different regions to obtain the final prediction.

In a word, comparing with traditional geometric and manual methods, the deep learning methods can extract deeper semantic features with higher efficiency and accuracy, and can be quickly applied to vessel segmentation tasks. However, the receptive fields in the above methods are fixed, which cannot fully extract the deep context information, besides, optimization problems still exist in training. In order to solve the problems mentioned above, our work has made the following improvements:

- A scale-aware dense residual module is proposed, which extracts multi-scale features of deep layers information by using dilated convolution group and dense residuals block.
- The UNet is combined with the structured residual convolution and attention gates to solve the optimization problem in training, suppress irrelevant information that affects segmentation, and highlight task related information.
- A multi-output weighted loss mechanism is proposed based on the deep supervision network. During the training process, each decoder layer is optimized by adding auxiliary network branches to accelerate the convergence process.

## Related work

The methods proposed in this paper are based on multi-scale feature extraction and aggregation, as well as the supervision network. Therefore, these two methods are briefly introduced here.

### Multiple scale feature extraction and aggregation

Multiple scale feature extraction and aggregation refers to using convolution of different receptive fields to extract features from the same input, and then merge. Deep network features lose more information after multiple down-samplings. Using multiple scale feature extraction and aggregation can more effectively preserve semantic information and restore image structure.

For the segmentation task of retinal blood vessels, compared with the segmentation of other organs with fixed structures, retinal vessels have a larger change in shape, and the vessels will show a longer distribution on the image. Therefore, it is one of the keys to improve the segmentation performance to fully understand the interaction between image regions.

The methods of enlarging receptive field include expanding the size of convolution kernel and using dilated convolution. Considering the computational cost, most methods choose to use dilation convolution to extract features at different scales through different dilation rates. For example, CE-Net [13] uses four channels of dilated convolutions with different numbers and receptive fields to achieve multiple scale feature extraction, to overcome the problem of semantic information loss by pooling layers, so as to capture more deep level features and provide more spatial information. Scs-Net [14] proposed a scale-aware feature aggregation module, which achieves multi-scale feature extraction through groups of dilated convolutions, and dynamically adjusts the receptive field by fusing the output features of adjacent dilated convolutions and subsequent weighting operations.

### Supervision network

**Fig. 1:**
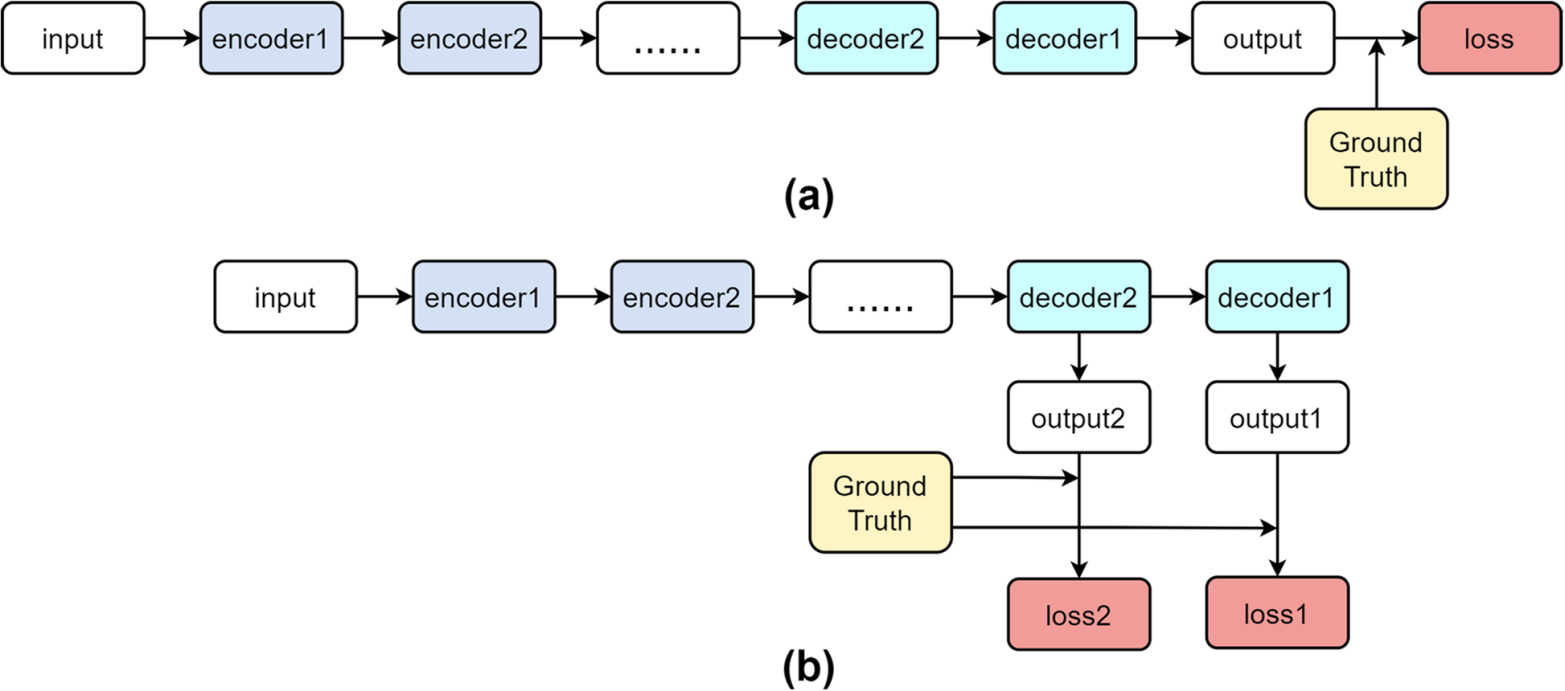
**a** shows the general flow of network computing loss, and **b** shows the general flow of network computing loss using deep supervision

The supervision network can make the network feedback in the training process to guide the network to optimize in a certain direction. One of the commonly used methods is deep supervision [15]. By supervising the backbone network, each decoder layer can be trained more fully to solve the problem of gradient or slow convergence. As shown in Fig. 1, Fig. 1(a) is a general network loss calculation process, only the output of the last decoder layer is compared with the real label to get the final loss. While Fig. 1(b) shows the process of using the deep supervision to calculate the loss, by outputting the feature map of each decoder layer, and comparing it with the ground truth, several different losses are obtained, and the final loss is weighted to guide the optimization of each decoder layer.

UNet++ [16] uses deep supervision mechanism to optimize the network by using a weighted loss function at the relay node and the decoder layer. UNet++ provides accurate mode and fast mode, the selection of the two modes determines the degree of model pruning and speed. UNet3+ [17] proposes a full scale deep supervision network to better learn features from full scale feature mapping. It generates an output at each decoder layer, after operations such as convolution and pooling, it gains losses by comparing with GroundTruth. ARU-GD [18] proposes a new guide decoder, which monitors the learning process of the decoder and helps to produce improved features, and proposes the weighted guidance loss to improve the prediction ability of each layer of the decoder, thus the prediction accuracy of the final layer is improved.

Therefore, the above methods inspire us to start from the direction of effectively processing the complex context information in the retinal vessel segmentation task, and strengthening the optimization of each decoder layer. By extracting multi-scale features and aggregating, using dynamic selection mechanism, we can learn more global semantic information, accelerate the convergence of the network, solve the optimization problem, and achieve more accurate segmentation.

## Methods

**Fig. 2:**
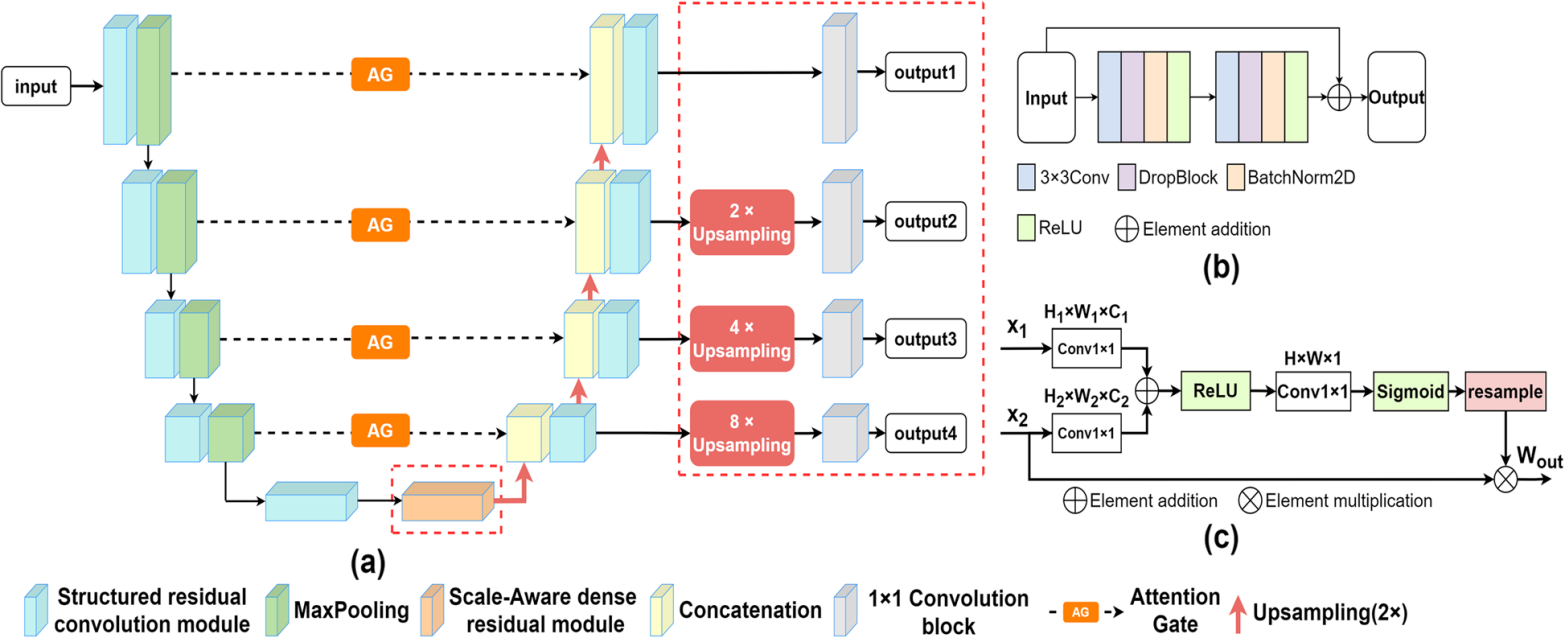
**a** represents the network structure proposed in this paper, the red dotted box represents the scale-aware dense residual module and multi-output weighted loss method, **b** represents the structured residual convolution module, and **c** represents the structure of the attention gate

The overall structure of the network proposed in this paper is shown in Fig. 2. The network adopts UNet structure, and its encoder layers and decoder layers are replaced with structured residual convolution modules, and the skip connections are replaced with attention gates. At the same time, the proposed scale-aware dense residual module is inserted between the encoder and decoder. Given an input image, the network first uses the encoder to extract features hierarchically. The output of the last layer of the encoder is transmitted to the scale-aware dense residual module, and then is transmitted to the decoder by upsampling. The decoder convolves the upsampled image to improve the geometric shape of the target. At the same time, the output of each decoder layer is separately compared with the Ground Truth to obtain the loss of each layer. Multi-output weighted losses are obtained by assigning different weight to different output losses, the output of the last decoder layer is used as the final prediction. The implementation details of the proposed scale-aware dense residual module, attention residual UNet and multi-output weighted loss are introduced below.

### Attention residual UNet

Convolutional neural network can improve the ability of feature extraction by stacking convolution layers. However, with the increase of depth, the difficulty of optimization will also increase. Gradient explosion and vanishing are easy to occur because of gradient layer by layer transmission. In order to solve the mentioned defects, the ordinary convolution is replaced with the residual convolution and residual UNet is obtained. Inspired by [5, 19], this paper uses a structured residual convolution module, whose structure is shown in the Fig. 2(b). Utilize additional skip to prevent gradient vanishing issues in deep network training, some previous methods used the Dropout layer to prevent the overfitting problem, but its characteristic of randomly discarding the activation units makes it unable to alleviate the overfitting problem, so DropBlock [20] is used instead of the Dropout layer. It effectively reduces the local spatial dependency of the convolutional network by discarding adjacent units, thereby enhancing the network’s generalization ability.

Although residual UNet can aggregate multi-scale features and solve gradient problems, the skip between encoder and decoder will transfer all information, include irrelevant background information. In order to reduce the impact of irrelevant factor on segmentation performance, a spatial attention gate [10] is introduced, attention gates restrain irrelevant areas during training, capture context information and highlight explicit features related to specific tasks, and avoid additional use of location models [11], the structure of the attention gate is shown in the Fig. 2(c). The attention gate has two inputs, one from the encoder layer and the other from the decoder layer. First, the number of channels is adjusted through the 1$\times$1 convolution layer respectively, then the adjusted two feature maps are merged. After the BatchNorm layer, the convolution layer reduces the number of channels to 1, the feature map passes through the Sigmoid function and multiplies with the input $X_2$ from the encoder to obtain the final weight matrix. The entire calculation process is as follows:

1 $$\begin{aligned} W_{out} = X_2 \otimes \delta \left( F_1\left( \sigma \left( F_1\left( X_1\right) \oplus F_1\left( X_2\right) \right) \right) \right) \end{aligned}$$

$\delta$ represents sigmoid, $\sigma$ represents ReLU, $F_1$ represents 1$\times$1 convolutional layer, $\otimes$ represents element multiplication and $\oplus$ represents element addition.

The network structure is shown in the Fig. 2(a), the part without the red dotted boxs is the attention residual UNet. In this paper, residual network and attention gates are combined for the following reasons: One is to handle gradient transfer situation in the optimization process, in this way, the network can stack more layers to make the training more stable; The other is to reduce the impact of irrelevant factors in the training process, highlight the features related to the task, so as to improve the segmentation accuracy. The ablation experiment results also show that the attention residual UNet has better performance than the single Attention U-Net and residual UNet.

### Scale-aware dense residual module

**Fig. 3:**
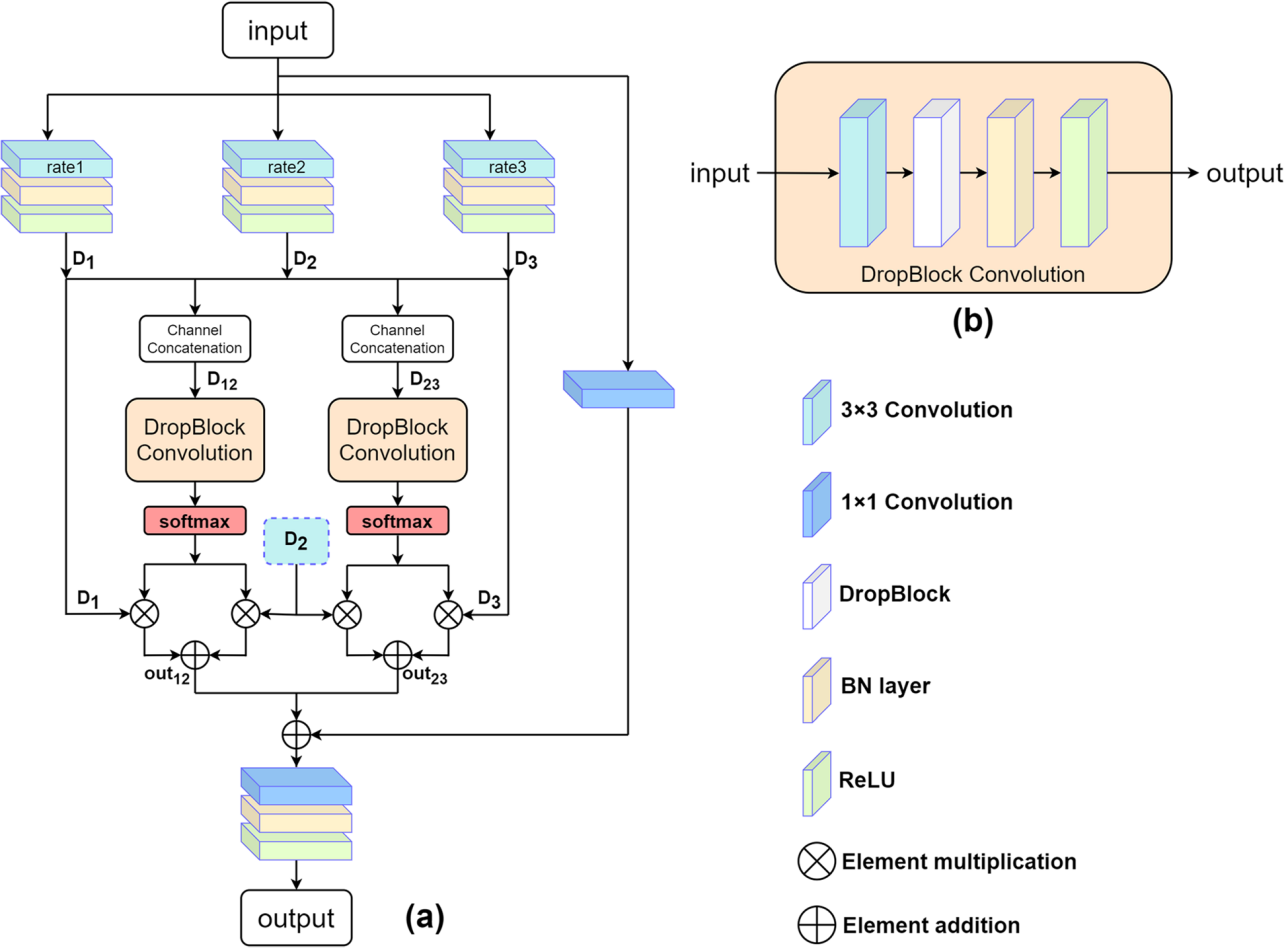
**a** shows the specific structure of scale-aware dense residual module, [rate1, rate2, rate3] represent different dilation rates of three dilated convolutions. **b** is DropBlock Convolution

Extracting multiple scale features is the key to improve accuracy, but large changes in retinal vessels images make the task difficult. Although UNet and some of its variants have the ability to extract features hierarchically, these models have the problem of fixed receptive fields, and vessels that do not match the size of the receptive fields will cause false segmentation or discontinuous segmentation. To solve the aforementioned defects, a scale-aware feature aggregation module is proposed in Scs-Net [14], which mainly includes two parts: multi-scale feature extraction and dynamic feature selection. In this paper, the proposed model combines SFA module and the residual dense network [9], BatchNorm layer and ReLU are added in front of dilated convolution. DropBlock [20], BatchNorm layer and ReLU are added on the basis of the convolution layer after merging features. The modification of the module is mainly to solve the following matters: first, BatchNorm layer can quicken the speed of convergence and prevent gradient problems; second, overfitting problems may occur in deep network training, ReLU and DropBlock can reduce the dependency between parameters and improve the generalization performance; third, the residual dense module has the function of feature reuse, compared with the separate convolution layer, it has a better ability to extract features, and can aggregate more information to improve the segmentation effect. Figure 3 shows the overall structure of the combination module.

The structure of multi-scale feature extraction can be calculated by the following methods. Suppose the input is represented by *X* with size of $H\times W\times C_{in}$, $C_{in}$ is the number of input channels, *H* and *W* represent the height and width of the input map respectively, and the output result is shown in Eq. 2:

2 $$\begin{aligned} D_i=\sigma \left( B\left( F_3 \left( X, rate \left( i\right) \right) \right) \right) , i=1,2,3 \end{aligned}$$

$D_i$ represents the output after the dilated convolution operation of the i-th path, *B* represents BatchNorm layer, and $\sigma$ represents ReLU function, $F_3$ represents 3 $\times$ 3 convolution operation, $rate\left( i\right)$ is the dilatation rate corresponding to the i-th channel dilated convolution, we set the dilatation rates of the three dilated convolutions to 1,3,5 based on [14]. Here, the number of output channels becomes $C_{out}$.

At the same time, a scale-aware mechanism is introduced into the model to automatically select the appropriate receptive field for feature map. For example, in the branch of the combined feature map of the dilated convolution module using rate1 and rate2 dilated rates in Fig. 3, the original SFA is used first 3 $\times$ 3 normal convolution, then uses ReLU and 1 $\times$ 1 convolution layer. In order to better apply it to the feature aggregation of the decoder layer and to transfer information to the next layer, this part of the original SFA is modified into a part of the transfer layer of the dense residual module. A 3 $\times$ 3 convolution layer is firstly used to adjust the channel, and then the feature maps with the number of channels after merging is adjusted to $C_{out}$, its height and width remain unchanged, meanwhile the DropBlock is introduced to prevent over fitting. The BatchNorm layer is used for standardization. Finally, the ReLU activation function undergoes nonlinear changes, as shown in Eq. 3:

3 $$\begin{aligned} D'_{12}=\sigma \left( B \left( D \left( F_3 \left( D_{12}, \theta \right) \right) \right) \right) \end{aligned}$$

where $\theta$ represents related parameters, and D represents DropBlock. This paper no longer uses the 1 $\times$ 1 convolution layer to adjust the channel, and keep the feature map shape as $H\times W\times C_{out}$, directly softmax the output of the DropBlock convolution module to generate two weight masks $\beta _{1}$ and $\beta _{2}$, the calculation dimension of softmax is set to the dimension of the channel. Multiply the weight mask with the $D_1$ and $D_2$ feature maps obtained previously, and then add the multiplied results, as shown in Eqs. 4 and 5:

4 $$\begin{aligned} \beta _{1}=\beta _{2}=softmax(D'_{12}) \end{aligned}$$

5 $$\begin{aligned} Out_{12}=\beta _{1}\otimes D_1\oplus \beta _{2}\otimes D_2 \end{aligned}$$

$\otimes$ represents the elements multiplication, $\oplus$ represents the elements addition. The final output of this path is denoted as $Out_{12}$, the calculation process of the other branch is the same, and the original input feature is passed through 1 $\times$ 1 convolution layer realizes the skip connetion for channel adjustment, as shown in Eq. 6:

6 $$\begin{aligned} X'=F_1\left( X,\theta \right) \end{aligned}$$

$F_1$ represents 1 $\times$ 1 convolution layer, the feature map $X'$ obtained from the original input through the skip adds with the output results of the two branches to get the final output results $Out_{final}$. As shown in Eq. 7:

7 $$\begin{aligned} Out_{final}=\sigma \left( B \left( F_1 \left( Out_{12} \oplus Out_{23}\oplus X' \right) \right) \right) \end{aligned}$$

To sum up, the modified scale-aware dense residual module can not only extract features of different scales through convolution modules with different dilatation rates, but also aggregate information of different scales with weights, and obtain stronger feature extraction and aggregation capabilities with dense residuals. At the same time, it can also adapt to the transmission structure of U-shape networks.

### Multi-output weighted loss

**Fig. 4:**
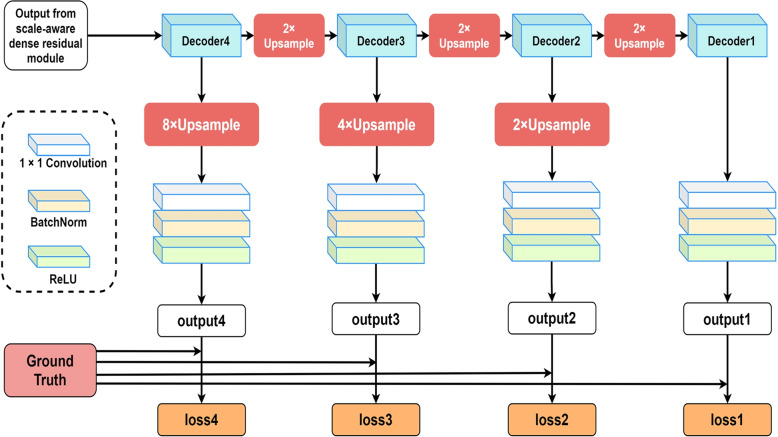
Process of Multi-output Weighted Loss

In ARU-GD [18], the output of each decoder layer is used to participate in the prediction of the final output, at the same time, inspired by the deep supervision networks used in UNet++ [16] and UNet3+ [17] , in order to fully utilize the segmentation results of each decoder layer, and fully consider the impact of feature maps at different levels on the final segmentation results, in this paper, additional operation branches will be added after each decoder layer. At the same time, weighted binary cross entropy loss function is used in each layer to alleviate the problem of imbalance between the front and background of the image, called multi-output weighted loss, which will help improve the accuracy of the network. The multi-output weighted loss structure is shown in Fig. 4. The output of each decoder layer needs to be upsampled to restore the size of the original input image. And we will use 1$\times$1 convolutional block instead of the classification network in [18] to adjust the number of channels for segmentation results. As shown in Eq. 8:

8 $$\begin{aligned} Out_i=\sigma \left( B\left( F_1\left( Up \left( D_i,factor\right) \right) \right) \right) , i=1,2,3,4 \end{aligned}$$

$F_1$ is 1 $\times$ 1 convolution layer, *B* is BatchNorm layer, $\sigma$ is ReLU, *Up* is the upsampling operation, $D_i$ indicates the i-th decoder layer, $Out_i$ indicates the output corresponding to the current layer, and *factor* indicates multiplier of upsampling. Refer to deep supervision, the total loss in this paper is calculated as shown in Eq. 9:

9 $$\begin{aligned} TotalLoss=\alpha \times \left( loss2+loss3+loss4\right) +\left( \beta -\alpha \right) \times loss1 \end{aligned}$$

The value of $\beta$ will be selected in implementation details. At this point, the previous layers will be assigned smaller weights, while the last layer will be assigned larger weights, the probability map of each layer passes through a fixed threshold to obtain binary segmentation output, but only the output of the last decoder layer is used as the final segmentation output.

In order to better allocate more appropriate weights to the foreground and background in the training process and accelerate the convergence of the network, we will use the weighted binary cross entropy loss function to calculate the output loss of each layer. The calculation formula is as follows:

10 $$\begin{aligned} Loss_{WBCE}=-\left[ w_1\cdot y\ln p+w_2\cdot \left( 1-y\right) \ln \left( 1-p\right) \right] \end{aligned}$$

*y* represents real label, *p* is the probability of the prediction category, and the value range is (0,1), $w_1$ and $w_2$ represent foreground weight and background weight respectively.

## Experiment

### Datasets

The datasets used in the experiment are the DRIVE and START. The DRIVE dataset contains 40 images, includes training set and test set, training set contains 20 retinal images with different brightness, and the number of images in test set is the same. The STARE dataset contains 20 labeled images, of which 5 are selected as the test set and the remaining 15 are used as the train set. Because there is less training data, data enhancement is required, such as horizontal and vertical flipping, and multi angle rotation such as 90 $^{\circ }$, 180 $^{\circ }$, 270 $^{\circ }$, etc, at the same time, Gaussian noise and salt pepper noise are added to improve the robustness of training results.

### Evaluation

The evaluation indicators used in this paper include dice coefficient, accuracy, mean Intersection over Union, and recall rate, among which dice coefficient and accuracy are the main indicators. Table 1 shows the calculation methods of the four evaluation indicators.

**Table 1:** Evaluation indicators and calculation formula

Indicator	Calculation formula
Dice coefficient	${2TP}\over {2TP+FP+FN}$
Accuracy	${TP+TN}\over {TP+TN+FP+FN}$
mIoU(binary classification)	$\frac{TP}{2(TP+FP+FN)} + \frac{TN}{2(TN+FP+FN)}$
Recall rate	$TP\over {TP+FN}$

### Implementation details

For better generalization performance, it is appropriate to set the training times epoch between 120 to 150 times. The number of batches is set to 2. The optimizer used for training is Adam, and the initial learning rate is 0.001. The weight set for multi-output weighted loss is $\alpha =0.125$ based on [18], for the DropBlock, the dropping probability is 0.18, and the size of the deleted block is 3. As for the selection results of the background and foreground weights of the weighted binary cross entropy loss function, the DRIVE dataset is taken as an experimental example. Table 2 shows the results. According to the comprehensive results, 0.8 for the foreground weight and 0.2 for the background weight are better.

**Table 2:** Results of binary cross entropy loss function with different weights

foreground weight	background weight	dice	acc	mIoU	recall
0.9	0.1	0.8027	**0.9665**	0.8175	**0.8815**
0.8	0.2	**0.8032**	**0.9665**	**0.8180**	0.8805
0.7	0.3	0.8004	0.9662	0.8156	0.8797
0.6	0.4	0.7973	0.9659	0.8134	0.8786
0.5	0.5	0.7950	0.9656	0.8116	0.8766

The experimental results of selecting $\beta$ values are shown in Table 3. According to the experimental results, it can be seen that the network performs better when the value of the $\beta$ is 1.0 on the DRIVE dataset and 0.9 on the STARE dataset.

**Table 3:** Test the values of $\beta$ on the DRIVE and STARE dataset

	DRIVE dataset				STARE dataset			
$\beta$	dice	acc	mIoU	recall	dice	acc	mIoU	recall
1.0	**0.8046**	**0.9660**	**0.8184**	0.8794	0.8281	0.9736	0.8397	0.8834
0.9	0.8016	0.9653	0.8131	0.8803	**0.8317**	0.9736	**0.8424**	0.8872
0.8	0.8026	0.9656	0.8169	**0.8831**	0.8306	**0.9738**	0.8415	0.8860
0.7	0.8008	0.9650	0.8055	0.8723	0.8286	0.9734	0.8399	0.8858
0.6	0.8044	0.9659	0.8158	0.8818	0.8315	0.9737	0.8421	**0.8899**
0.5	0.8003	0.9650	0.8149	0.8733	0.8278	0.9734	0.8392	0.8841

### Comparison of algorithm results

**Fig. 5:**
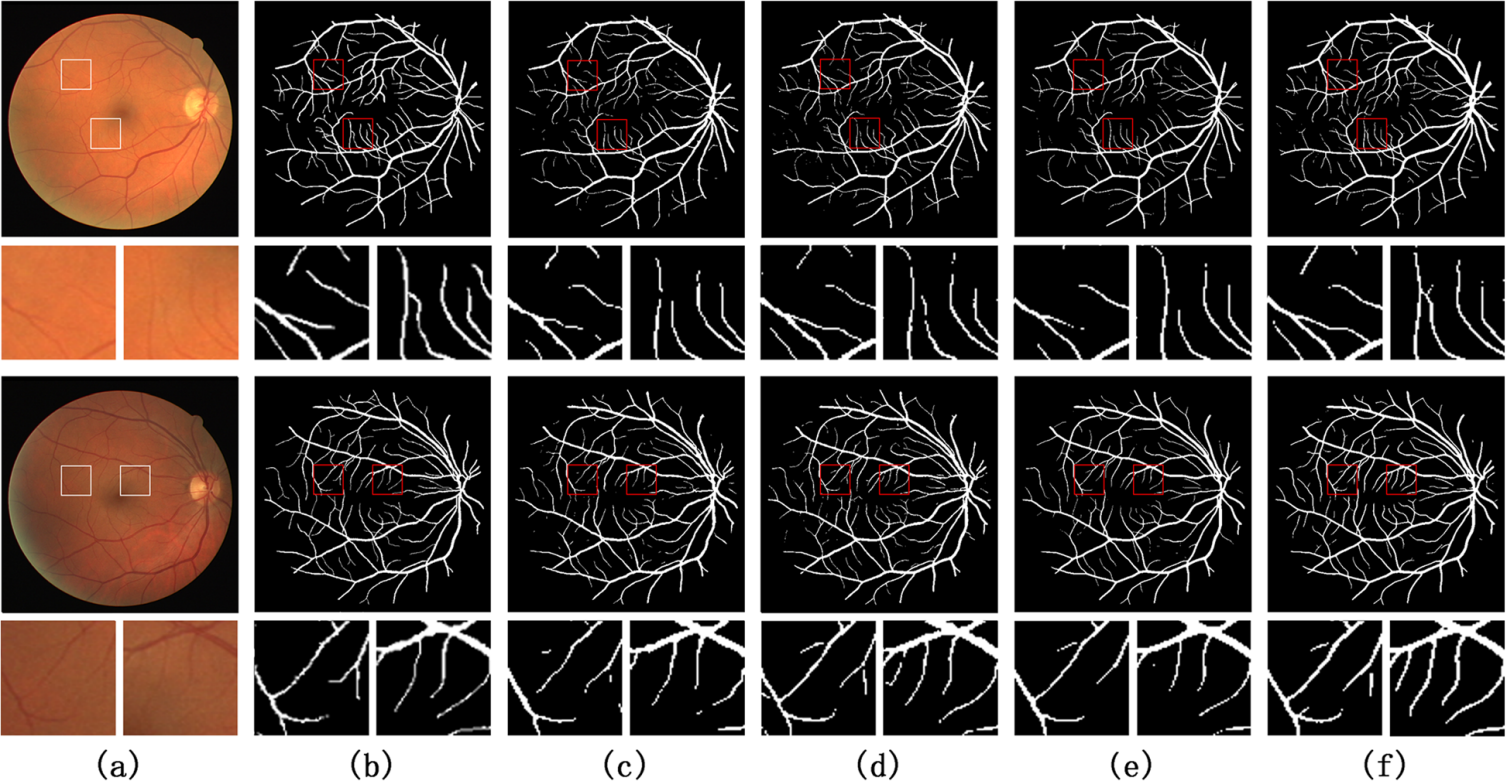
The result of segmentation on DRIVE dataset. The small graph corresponds to the selected area in the small box, marked by letters, respectively: **a** the original image, **b** Ground Truth, **c** UNet, **d** DR-Vnet, **e** CRAUnet, **f** the network proposed in this paper

**Fig. 6:**
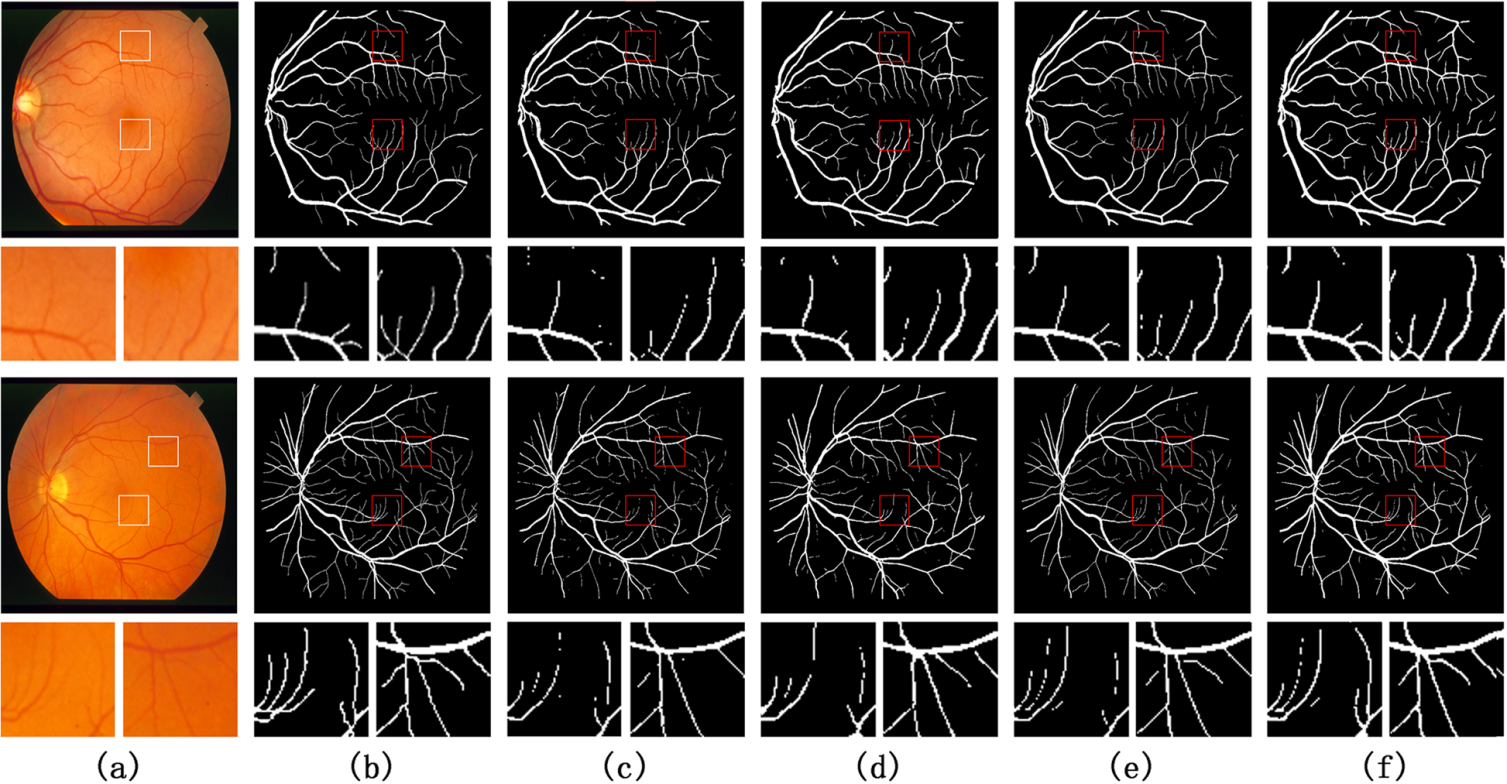
The result of segmentation on the STARE dataset. The small graph corresponds to the selected area in the small box, marked by letters, respectively: **a** the original image, **b** Ground Truth, **c** UNet, **d** DR-Vnet, **e** CRAUnet, **f** the network proposed in this paper

The proposed network is tested on DRIVE and STARE datasets, and compared with the latest methods, all methods are compared on the same test sets. Figure 5 shows the experimental results of DRIVE dataset. The methods in Fig. 5(c) to (e) correspond to UNet, DR-Vnet, and CRAUnet respectively. Figure 5(f) is the experimental results corresponding to the methods proposed in this paper. Figure 6 shows the segmentation results on the STARE dataset. As for the parts marked with small red boxes in the segmentation results (c) to (f) in Figs. 5 and 6, other methods have lost more details, and the proposed method can better protect the capillaries part, more close to the segmentation of the Ground Truth, to a certain extent, reduce the missed segmentation or false segmentation.

The comparison results of evaluation indicators are shown in Table 4. On the DRIVE data set, compared with the DR-Vnet [9], the four indicators increased by 0.29%, 0.02%, 0.49% and 0.28% respectively, and compared with the CRAUNet [21], the other three indicators increased by 0.12%, 0.28% and 0.47% respectively, except for the lower accuracy. On the STARE dataset, compared with the DR-Vnet, the four indicators increased by 0.34%, 0.06%, 0.19% and 0.4% respectively, and compared with the CRAUNet, the other two indicators increased by 0.16% and 0.29% respectively, except for the lower accuracy and mIoU. Therefore, the proposed network has a better segmentation performance.

**Table 4:** Comparison of segmentation performance of different networks on different datasets

	DRIVE dataset				STARE dataset			
network	dice	acc	mIoU	recall	dice	acc	mIoU	recall
UNet [4]	0.7751	0.9622	0.7963	0.8661	0.8103	0.9705	0.8251	0.8693
Attention U-Net [10]	0.7849	0.9644	0.8044	0.8720	0.8158	0.9718	0.8297	0.8769
CE-Net [13]	0.7794	0.9630	0.7996	0.8678	0.8141	0.9711	0.8280	0.8737
SA-Unet [5]	0.7993	0.9654	0.8120	0.8783	0.8284	0.9731	0.8395	0.8892
Sine-Net [22]	0.8006	0.9665	0.8167	0.8757	0.8303	0.9738	0.8414	0.8858
DR-Vnet [9]	0.8011	0.9665	0.8165	0.8782	0.8307	0.9733	0.8419	0.8844
CRAUnet [21]	0.8028	**0.9681**	0.8186	0.8763	0.8325	**0.9746**	**0.8441**	0.8855
Our network	**0.8040**	0.9667	**0.8214**	**0.8810**	**0.8341**	0.9739	0.8438	**0.8884**

### Ablation experiment

In order to verify the contribution of different methods proposed in this paper to network improvement, ablation experiments were conducted on these modules. Take DRIVE dataset as an example, first verify the improvement of the combined network of residual UNet and attention gates, Table 5 shows that the four indicators of Att-Res UNet have respectively increased by 1.08%, 0.12%, 0.78% and 0.77% compared with Attention U-Net, and 0.88%, 0.05%, 0.64% and 0.57% compared with Res-UNet. Then, on the basis of Att-Res UNet, verify the performance improvement of scale-aware dense residual module and multi-output weighted loss respectively, according to Table 5, the four indicators with scale-aware dense residual module increased by 0.39%, 0.01%, 0.28% and 0.46% respectively compared with Att-Res UNet, and the network with multi-output weighted loss increased by 0.65%, 0.10%, 0.51% and 0.05% respectively. It can be seen that the network with multi-output weighted loss improved more significantly. Finally, combine Att-Res UNet with SDR and multi-output weighted loss to get the final network, compared with the Att-Res UNet only added with SDR, the indicators increased by 0.43%, 0.16%, 0.42% and 0.42% respectively, and by 0.17%, 0.07%, 0.19% and 0.83% respectively compared with the Att-Res UNet only added with multi-output weighted loss. And the results of the ablation experiment on the STARE dataset are shown in Table 5.

**Table 5:** Results of ablation experiments on DRIVE and STARE dataset, where Res-UNet represents UNet that replaces all the original convolution modules with structured residual convolution modules, Att-Res UNet represents Attention residual UNet, SDR represents scale-aware dense residual module and ML represents multi-output weighted loss

	DRIVE dataset				STARE dataset			
Module	dice	acc	mIoU	recall	dice	acc	mIoU	recall
Attention U-Net [10]	0.7851	0.9638	0.8041	0.8728	0.8130	0.9717	0.8293	0.8737
Res-UNet	0.7871	0.9645	0.8055	0.8748	0.8184	0.9721	0.8319	0.8789
Att-Res UNet	0.7959	0.9650	0.8119	0.8805	0.8256	0.9733	0.8376	0.8809
Att-Res UNet+SDR	0.7998	0.9651	0.8147	0.8851	0.8299	0.9736	0.8409	0.8877
Att-Res UNet+ML	0.8024	0.9660	0.8170	0.8810	0.8316	0.9739	0.8423	0.8864
Att-Res UNet+SDR+ML	0.8041	0.9667	0.8189	0.8893	0.8350	0.9744	0.8445	0.8920

## Conclusion

In this paper, a new retinal vessel segmentation network is proposed. The structured residual convolution module is used in the encoder to obtain the feature information and approximate position information of the image, after the last encoder layer, the scale-aware dense residual module is used for multi-scale feature extraction and aggregation, the decoder also uses structured residual convolution module to collect semantic information and feature maps, we uses attention gates to suppress irrelevant background features and further strengthen relevant target features in the training process, uses multi-output weighted loss to independently predict and compare the output of each decoder layer, generates better feature representation in each layer, shifts the weighted loss layer by layer, and helps improve the model segmentation accuracy. Then the feasibility of the network in this paper is verified by comparing with the latest methods on DRIVE and STARE datasets.

Although the network proposed in this paper can extract more continuous and complete capillaries to a certain extent, there are still some shortcomings, for example, when the contrast between the foreground and background of the image is too low, it is difficult for the network to distinguish between the target and the background, and there will be some false segmentation or missing segmentation. The following research will consider the contrast to improve the ability of network feature extraction.

## Data Availability

The datasets used in our research are public available. The datasets generated and/or analysed during the current study are available in the DRIVE and STARE repository, web link: (https://drive.grand-challenge.org/) and (https://cecas.clemson.edu/ahoover/stare/).
